# Supplementary figures and images for: Plasmid analysis of NDM metallo-β-lactamase-producing Enterobacterales isolated in Vietnam
Source: PLoS One. 2021 Jul 28;16(7):e0231119. doi: 10.1371/journal.pone.0231119 (PMC8318238; doi:10.1371/journal.pone.0231119)

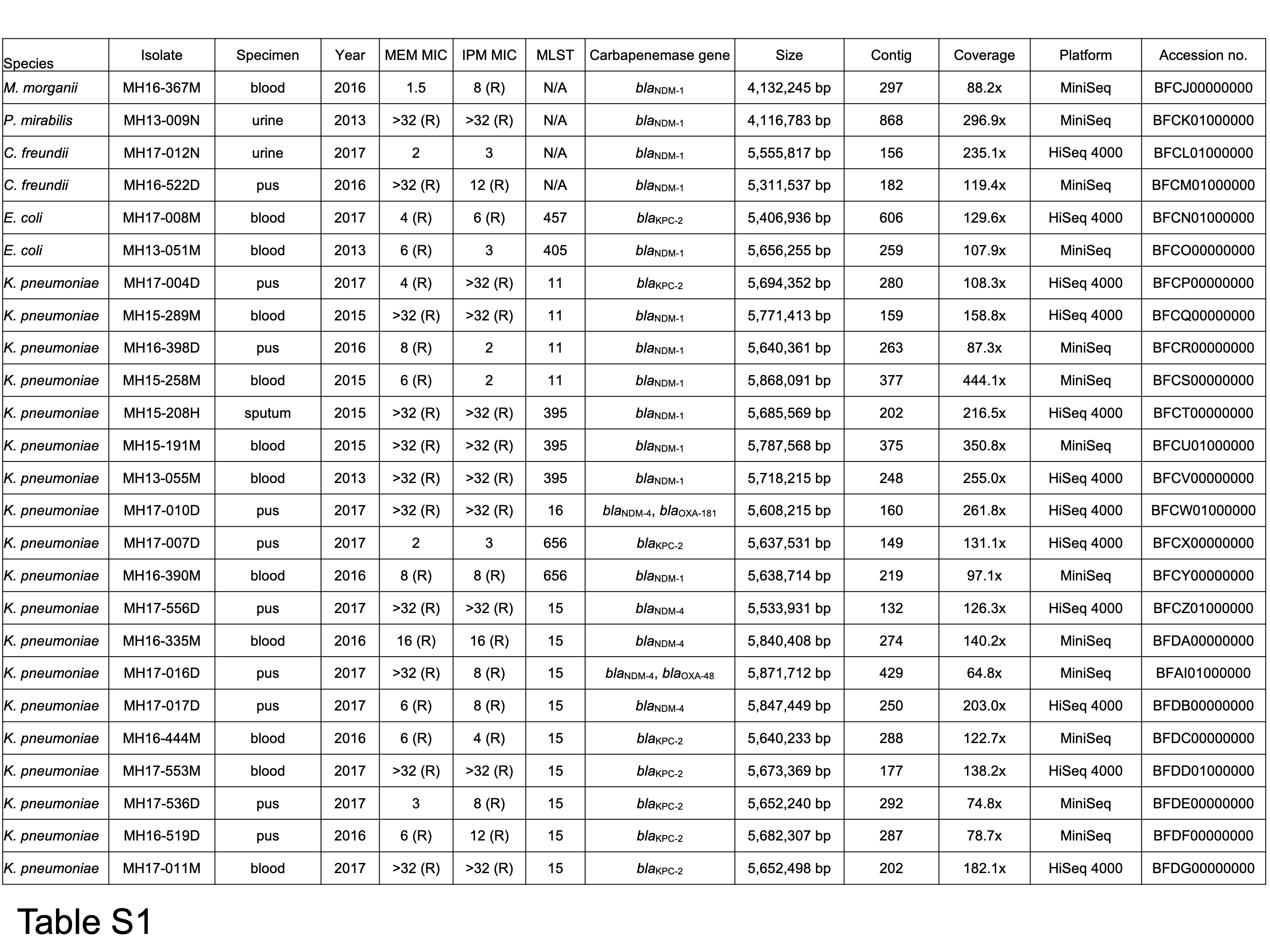

Supplement: S1 Table — Also, sequence types of multilocus sequence typing (MLST) analysis determined from genomes, carbapenemase genes detected by ResFinder in genomes, sizes and contigs of genomes, coverages in short-read sequencing, Illumina sequencing platforms, and accession numbers of genomes are shown. According to the CLSI 2020 guidelines, Breakpoints of meropenem and imipenem are as follows: ≤1 μg/mL, susceptible; 2 μg/mL, intermediate; ≥4 μg/mL, resistant (R). (TIFF) [file pone.0231119.s001.tiff]

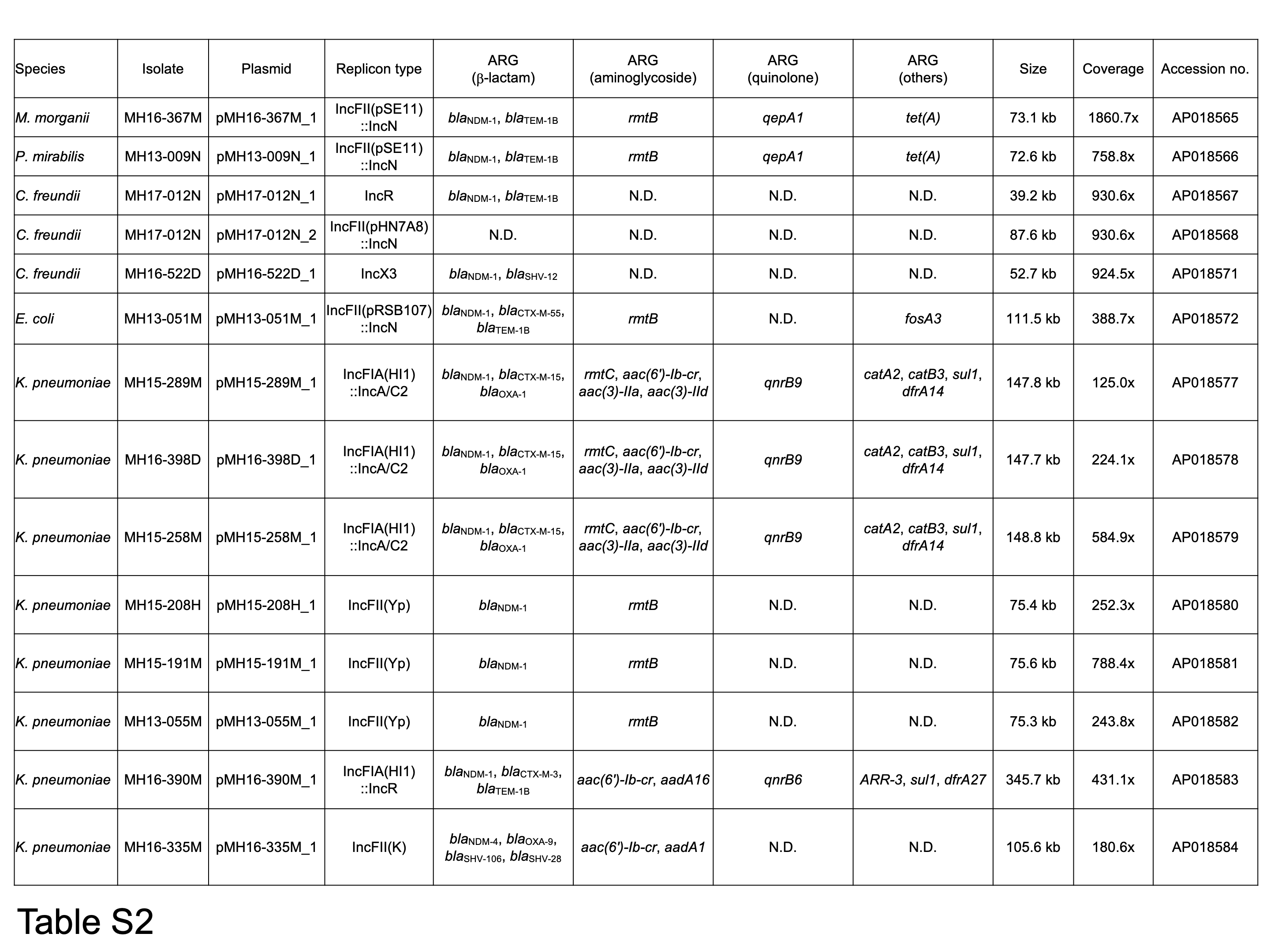

Supplement: S2 Table — Also, replicon types detected by PlasmidFinder and antimicrobial resistance genes (ARGs) detected by ResFinder in plasmids, sizes of plasmids, and coverages in long-read sequencing, and accession numbers of plasmids are shown. (TIFF) [file pone.0231119.s002.tiff]
